# Targeting chemoresistant colorectal cancer via systemic administration of a BMP7 variant

**DOI:** 10.1038/s41388-019-1047-4

**Published:** 2019-10-07

**Authors:** Veronica Veschi, Laura R. Mangiapane, Annalisa Nicotra, Simone Di Franco, Emanuela Scavo, Tiziana Apuzzo, Davide S. Sardina, Micol Fiori, Antonina Benfante, Maria L. Colorito, Gianfranco Cocorullo, Felice Giuliante, Calogero Cipolla, Giuseppe Pistone, Maria Rita Bongiorno, Aroldo Rizzo, Courtney M. Tate, Xiaohua Wu, Scott Rowlinson, Louis F. Stancato, Matilde Todaro, Ruggero De Maria, Giorgio Stassi

**Affiliations:** 1 Department of Surgical, Oncological and Stomatological Sciences (DICHIRONS), University of Palermo, 90127 Palermo, Italy; 2 Department of Oncology and Molecular Medicine, Istituto Superiore di Sanità, Rome, Italy; 3 Istituto di Patologia Generale, Università Cattolica del Sacro Cuore, 00168 Rome, Italy; 4 Fondazione Policlinico Universitario A. Gemelli—I.R.C.C.S., 00168 Rome, Italy; 5 Department of Health Promotion Sciences, Internal Medicine and Medical Specialties (PROMISE), University of Palermo, 90127 Palermo, Italy; 6 Pathology Unit, Ospedali Riuniti Villa Sofia-Cervello, Palermo, Italy; 7 Lilly Research Laboratories, Lilly Corporate Center, Indianapolis, IN 46285 USA

**Keywords:** Colorectal cancer, Cancer stem cells

## Abstract

Despite intense research and clinical efforts, patients affected by advanced colorectal cancer (CRC) have still a poor prognosis. The discovery of colorectal (CR) cancer stem cell (CSC) as the cell compartment responsible for tumor initiation and propagation may provide new opportunities for the development of new therapeutic strategies. Given the reduced sensitivity of CR-CSCs to chemotherapy and the ability of bone morphogenetic proteins (BMP) to promote colonic stem cell differentiation, we aimed to investigate whether an enhanced variant of BMP7 (BMP7v) could sensitize to chemotherapy-resistant CRC cells and tumors. Thirty-five primary human cultures enriched in CR-CSCs, including four from chemoresistant metastatic lesions, were used for in vitro studies and to generate CR-CSC-based mouse avatars to evaluate tumor growth and progression upon treatment with BMP7v alone or in combination with standard therapy or PI3K inhibitors. BMP7v treatment promotes CR-CSC differentiation and recapitulates the cell differentiation-related gene expression profile by suppressing Wnt pathway activity and reducing mesenchymal traits and survival of CR-CSCs. Moreover, in CR-CSC-based mouse avatars, BMP7v exerts an antiangiogenic effect and sensitizes tumor cells to standard chemotherapy regardless of the mutational, MSI, and CMS profiles. Of note, tumor harboring *PIK3CA* mutations were affected to a lower extent by the combination of BMP7v and chemotherapy. However, the addition of a PI3K inhibitor to the BMP7v-based combination potentiates *PIK3CA*-mutant tumor drug response and reduces the metastatic lesion size. These data suggest that BMP7v treatment may represent a useful antiangiogenic and prodifferentiation agent, which renders CSCs sensitive to both standard and targeted therapies.

## Introduction

Advanced colorectal cancer (CRC) is still a major challenge for clinical oncologists, being among the top causes of cancer-related death worldwide [1]. Cancer stem cells (CSCs) are key players in tumor initiation and development of metastasis [2, 3]. In recent years, many studies investigating the biological behavior of CSCs inspired the design of innovative therapeutic strategies for CRC. Genetic and epigenetic changes, overexpression of antiapoptotic proteins, and enhanced DNA repair machinery define the common traits of CSCs [4, 5]. The acquisition of an epithelial–mesenchymal transition (EMT) phenotype confers to CSCs the ability to invade and metastasize [6]. Among the most studied CSC markers, CD133 has been reported to identify CR-CSCs [7]. More recently, we determined that a splicing variant of CD44, CD44v6, is a functional marker expressed in CR-CSCs able to migrate and engraft at distant sites [8]. In line with the enhanced Wnt signaling observed in CR CD44v6^+^ cells, β-catenin activation induces CD44v6 expression in CR-CSCs. This pathway is sustained by the activation of the PI3K/AKT pathway, which promotes β-catenin activation through the inhibition of GSK3β, a key component of its destruction complex [8]. According to the multistep model, the progressive acquisition of mutations in proto-oncogenes or tumor suppressor genes defines specific stages of CRC [9]. Aberrant alterations of principal components of pathways involved in intestinal stem cell self-renewal endorse the disruption of intestinal niche equilibrium [10]. In addition to Wnt, Notch, and Sonic hedgehog pathways, bone morphogenetic proteins (BMPs) finely regulate the intestinal niche homeostasis balancing self-renewal and differentiation [11, 12]. BMPs are members of the TGF-β superfamily and regulate many fundamental biological processes during development. BMPs bind both type I and type II receptors (BMPR1A, BMPR1B, and BMPR2) to achieve a variety of cellular functions [13]. The activation of this pathway promotes the phosphorylation of SMAD1, 5, and 8 that in association with SMAD4 regulates the expression of genes involved in the differentiation process [14, 15]. BMP antagonists (gremlin and noggin) tightly modulate BMPs activity [16]. In healthy colon mucosa, the expression of BMPs and their antagonists is polarized. BMPs are mainly located at the top, while BMP antagonists at the base of colon crypt [17]. Alterations of BMP pathways can imbalance the homeostasis of the intestinal stem cell niche, thus favoring the development and progression of CRC. Indeed, the loss of BMPR2 and SMAD4 expression has been reported in sporadic CRC, whereas germline mutations of *BMPR1* and *SMAD4* genes have been demonstrated to enhance the susceptibility to develop juvenile polyposis, supporting that TGF-β signaling inactivation plays a key role in CRC development [18–22]. In intestinal stem cells, BMP signaling counteracts the Wnt pathway activity by impairing the nuclear accumulation of β-catenin through a PTEN-dependent AKT inhibition [23]. This antagonistic activity of BMP signaling against stem cells and Wnt pathway seems preserved in the cancer counterpart as indicated by the ability of BMP4 to promote differentiation and apoptosis of CR-CSCs [24].

BMP expression varies across tumor subtypes [25]. BMP7 is widely expressed in many tumors including breast, prostate, and colon cancer, and it is implicated in the regulation of cell proliferation [26–28]. However, its functional association with tumorigenicity and metastasis formation is still poorly understood. Recently, a human variant of BMP7 with enhanced stability and solubility (BMP7v) has been developed, by introducing mutations into the N terminus of BMP7 prodomain [29]. In glioblastoma stem-like cells, BMP7v impairs their proliferation and invasive capability by inducing differentiation [30] and significantly decreases angiogenesis. BMP7v, unlike BMP7, is not recognized by most of the BMP endogenous antagonists, such as noggin, gremlin, chordin, and chordin-like 2, due to reduced binding [31]. Disease progression in CRC is mostly due to the emergence of chemoresistant CSCs after therapeutic interventions [32]. Different mechanisms and biomarkers have been proposed so far to study and predict chemoresistance. Both microsatellite instability (MSI) and consensus molecular subtype (CMS) profiles correlate with the chemotherapy response in CRC. Specifically, MSI CRCs have been correlated with a better prognosis [33] but also with a lack of benefit from oxaliplatin (oxa) plus 5-fluorouracil (5-FU) therapy [34, 35]. CMS2 CRC is as the subset that most benefits from the chemotherapy, while the CMS4 results resistant to conventional therapy [36, 37]. We demonstrated that the activation of the PI3K/AKT pathway is essential for preserving the stem cell status in CRC CD44v6^+^ cells [8]. PI3K activation results in the onset of alternative signaling pathways, including Wnt-β-catenin axis activation that promotes CR-CSC survival, invasion, and development of metastases [38]. Using the BMP7v, here we have studied the possibility of targeting chemoresistant CRC through the induction of CSC differentiation. We provide evidence supporting the use of BMP7v in combination with chemotherapeutic compounds and/or PI3K inhibitors for CRC treatment.

## Results

### BMP7 is highly expressed in low-grade CRC patients

**Fig. 1:**
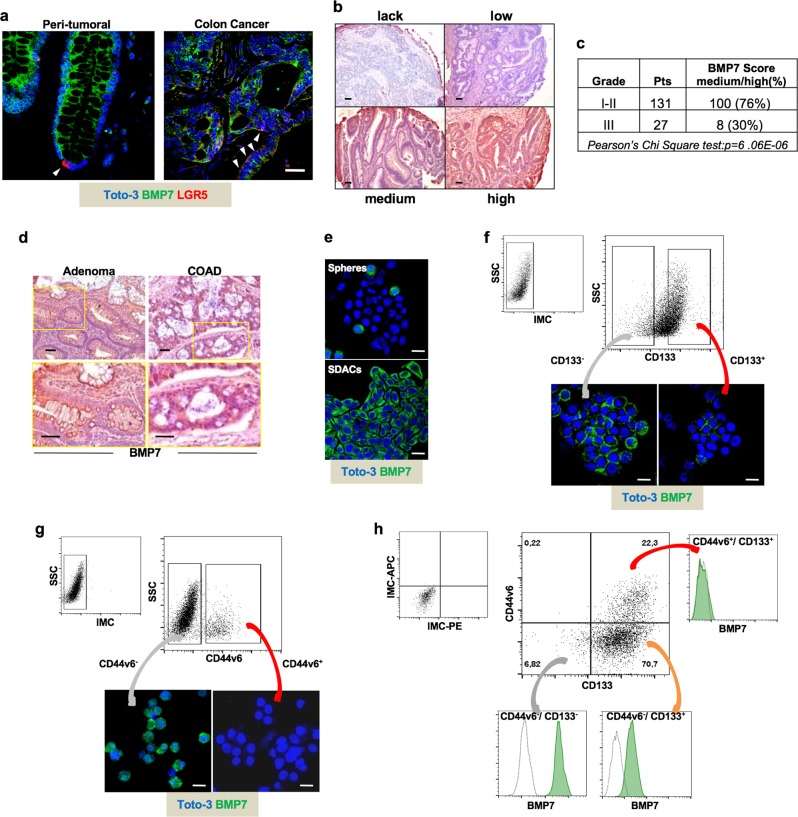
BMP7 is confined to differentiated CRC cells. **a** Immunofluorescence analysis of BMP7 (green color) and LGR5 (red color) on peritumoral mucosa and colon cancer paraffin-embedded tissues performed on CSC#8. One representative tumor from twenty different tumors examined is shown. Nuclei were counterstained by Toto-3 (blue color). White arrowheads indicate LGR5^+^ cells at the base of colon crypt. The scale bar represents 100 µm. **b** Immunohistochemical analysis of BMP7 on CRC TMAs in lack, low, medium, and high staining intensity (red color). Nuclei were counterstained by aqueous hematoxylin (blue color). The scale bar represents 100 µm. **c** Association of BMP7 expression with score medium/high and the pathological grading in CRC TMAs provided by TRISTAR technology group. **d** Immunohistochemical analysis of BMP7 (red color) in paraffin-embedded sections of colon adenomas and adenocarcinoma (COAD). Nuclei were counterstained by aqueous hematoxylin (blue color). The scale bar represents 100 µm. **e** Immunofluorescence analysis of BMP7 (green color) in CRC sphere cells and their differentiated progeny SDACs. One representative of fifteen different CR-CSC lines (CSC#1–3, 5–7, 10,11, 14–16, 18, 25, 33, and 40) is shown. Nuclei were counterstained by Toto-3 (blue color). The scale bars represent 20 µm. **f** Representative flow cytometry analysis of CD133 in CRC sphere cells and its relative isotype-matched control (IMC) (upper panels) performed on CSC#4, 8, and 23–26. Immunofluorescence analysis of BMP7 (green color) in CD133^+^ and CD133^−^ enriched CRC sphere cell subpopulations (lower panels). Nuclei were counterstained by Toto-3 (blue color). The scale bars represent 20 µm. **g** CD44v6 expression profiles of cells as described in **f** (upper panels). Expression of BMP7 (green color) in CD44v6^+^ and CD44v6^−^ enriched CRC sphere cell subpopulations assessed by immunofluorescence analysis (lower panels). Nuclei were counterstained by Toto-3 (blue color). The scale bars represent 20 µm. **h** Flow cytometry analysis of BMP7 (green histograms) in enriched CD44v6^−^/CD133^−^, CD44v6^−^/CD133^+^, and CD44v6^+^/CD133^+^ CRC subpopulations performed as shown in **f**. Dotted line histograms indicate the relative IMC

In accordance with the current literature, we found BMP7 abundantly expressed in CRC tissues, compared with peritumoral mucosa (Fig. 1a). BMP7 expression was limited to the apical part and absent in the LGR5^+^ stem cells located at the very base of the cancer gland (Fig. 1a, left panel). Analysis of a cohort of 158 CRC patients showed a significant correlation between medium/high BMP7 expression and the low-grade (I-II) tumors, which was confirmed by the analysis of a cohort of CRC in R2 database (Fig. 1b, c and Supplementary Fig. 1a). Interestingly, BMP7 was found highly expressed in both colon adenoma and adenocarcinoma, suggesting this phenomenon as an early event in cancer (Fig. 1d). In line with the expression of BMP7 in the differentiated part of the colon gland, BMP7 was remarkably expressed in sphere-derived adherent cells (SDACs), while it was present in few cells across CRC spheres, which are enriched in stem-like cells (Fig. 1e). Moreover, we found that CD133^-^ cells showed a higher percentage of BMP7-expressing cells as compared with the CD133^+^ compartment (Fig. 1f and Supplementary Fig. 1b, c). Interestingly, CD44v6^+^ cells lacked BMP7 expression, which was conversely confined to the CD44v6^-^ cell compartment (Fig. 1g and Supplementary Fig. 1d, e). In accordance with the immunofluorescence studies, flow cytometry analysis showed that BMP7 is expressed in CD133^−^/CD44v6^−^ cells and in a fraction of CD133^+^ cell compartment, whereas it is nearly undetectable in enriched CD44v6^+^/CD133^+^ stem-like cells (Fig. 1h). These data demonstrate that BMP7 is predominantly expressed in differentiated CRC cell population, particularly in low-grade CRCs.

### BMP7v affects CD44v6 expression in CR-CSCs

**Fig. 2:**
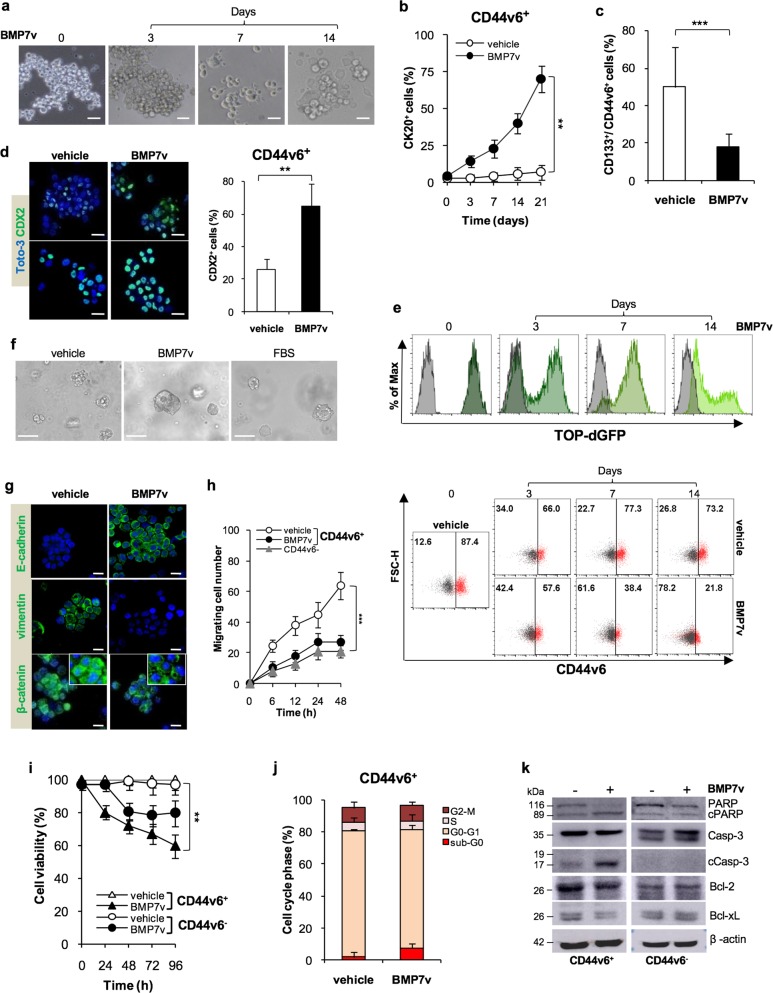
BMP7v treatment promotes CR-CSC differentiation. **a** Phase-contrast microscopy analysis of CD44v6^+^ CRC sphere cells treated with BMP7v at the indicated time points. One representative of CSC#1, 2, 4, 5, 7, and 23–26 is shown. The scale bar represents 20 µm. **b** Percentage of CK20 positive cells in CD44v6^+^ CR-CSCs treated with vehicle or BMP7v up to 21 days evaluated by immunofluorescence analysis. Data are expressed as mean ± SD of experiments performed in 15 CRC sphere cell lines (CSC#1–3, 5–7, 10,11, 14–16, 18, 25, 33, and 40). **c** Flow cytometry analysis of CD133/CD44v6 on CRC sphere cells treated with vehicle or BMP7v for 14 days. Data reported are mean ± SD of 15 CRC sphere cell lines analyzed (CSC#1–8, 10,11, 14–16, 18, and 25). **d** (left panels) Immunofluorescence analysis of CDX2 on CR-CSCs upon 14 days of BMP7v treatment. One representative of CSC# 3, 9, and 21 is shown. Nuclei were stained with Toto-3 (blue color). The scale bars represent 20 µm. (right panel) Percentage of CDX2 positive cells in CD44v6^+^ CR-CSCs treated with vehicle or BMP7v up to 14 days evaluated by immunofluorescence analysis. Data are expressed as mean ± SD of experiments performed in CSC# 3, 9, and 21. **e** Flow cytometry analysis of TOP-dGFP or CD44v6 in enriched CD44v6^+^ sphere cells treated with BMP7v up to 14 days. One representative experiment of CSC#1, 2, 4, 7, and 10 is shown. **f** Phase-contrast microscopy analysis of TOP-dGFP CRC sphere cells grown in matrigel drops and treated with vehicle, BMP7v or FBS for 14 days. One representative of CSC# 8, 9, and 11 is shown. The scale bar represents 100 µm. **g** Immunofluorescence analysis of E-cadherin, vimentin, and β-catenin (green color) in CD44v6^+^ CRC cells exposed to vehicle or BMP7v for 14 days. One representative experiment performed in cells as in e is shown. Nuclei were stained with Toto-3 (blue color). The scale bars represent 20 µm. **h** Migrating CD44v6^+^ and CD44v6^−^ cells treated with vehicle or BMP7v up to 48 h. Data are shown as mean ± SD of three independent experiments performed in five CRC sphere cell lines (CSC#1, 5, 7, 10, and 12). **i** Cell viability percentage of enriched CD44v6^+^ and CD44v6^−^ cells treated with vehicle or BMP7v up to 96 h. Data are shown as mean ± SD of different experiments performed in CSC#1, 2, 4, 7, and 10. **j** Cell cycle analysis in CD44v6^+^ CR-CSCs exposed to vehicle or BMP7v for 72 h. The data show percentage of cell number in sub-G0, G0/G1, S, and G2/M phases. Data are expressed as mean ± SD of three independent experiments performed in five different CRC sphere cell lines as in **e**. **k** Immunoblot analysis of PARP, cleaved PARP (cPARP), Caspase-3 (Casp-3), cleaved Caspase-3 (cCasp-3), Bcl-2, Bcl-xL in CD44v6^+^, and CD44v6^−^ enriched cells treated as in **e** for 72 h. β-actin was used as loading control. One representative experiment performed in three different CRC sphere cell lines (CSC#1, 4, and 7)

In order to define the potential of BMP7 as a prodifferentiation agent, we next evaluated whether the BMP signaling pathway components were conserved in a CRC model. We observed that type I and type II BMP receptors are expressed in both CD44v6^+^ and CD44v6^−^ fraction with more pronounced expression levels of BMPR2 in the CD44v6^−^ counterpart (Supplementary Fig. 2a, b). In order to investigate the effects of BMP7 on CR-CSCs, we used a modified BMP7 with enhanced stability and solubility (BMP7v) as previously described [30]. The dose concentration of 100 ng/ml of BMP7v corresponded to the IC50 and was also preferred for the in vitro ability to inhibit the colony forming capacity of CR-CSCs even in the presence of high doses of BMP antagonists, gremlin, and noggin (Supplementary Fig. 2c, d). CD44v6^+^ cells exposed to BMP7v up to 21 days displayed a gradual morphological differentiation (Fig. 2a), paralleled by the acquisition of CK20 expression (Fig. 2b). In accordance, BMP7v significantly reduced the percentage of cells expressing the CD133/CD44v6 CSC markers and increased the number of CDX2^+^ cells within CRC spheres (Fig. 2c, d and Supplementary Fig. 2e, f). In a cohort of CRC patients, expression levels of CDX2 are inversely correlated with tumor grading (Supplementary Fig. 2g).

Interestingly, flow cytometry analysis of primary CRC sphere cells transduced with a β-catenin/TOP-dGFP reporter lentiviral vector showed that exposure to BMP7v was able to progressively decrease β-catenin activity and CD44v6 expression (Fig. 2e and Supplementary Fig. 2h). Moreover, BMP7v treatment caused the reduction of β-catenin activity and differentiation of CRC organoid, which was highlighted by lumen formation and cell polarization (Fig. 2f and Supplementary Fig. 2i). In the presence of BMP7v, CD44v6^+^ CRC sphere cells acquired E-cadherin expression, displayed loss of vimentin and reduction of nuclear β-catenin (Fig. 2g). A large cohort of CRC patients showed a positive correlation between the expression levels of BMP7 and E-cadherin (*CDH1*) (Supplementary Fig. 3a). In agreement, xenograft tumors generated by the injection of CR-CSCs (CSC# 1, 8, and 25), displayed a major number of cells expressing E-cadherin after BMP7v treatment (Supplementary Fig. 3b). Accordingly, the in vitro invasive capacity of CD44v6^+^ CR-CSCs was significantly impaired following treatment with BMP7v (Fig. 2h). Following 96 h of treatment, CD44v6^+^ fraction exhibited a more pronounced sensitivity to BMP7v-induced cell death than CD44v6^−^ cells (Fig. 2i). Although BMP7v treatment did not significantly affect G0/G1 to G2/M phase transition, it enhanced to a small extent the sub-G0 phase in CD44v6^+^ CR-CSCs (Fig. 2j and Supplementary Fig. 3c). In line with the induction of cell death, BMP7v treatment induced activation of both PARP and Caspase-3 and a downregulation of Bcl-2 and Bcl-xL in CD44v6^+^ cells (Fig. 2k and Supplementary Fig. 3d, e). These data indicate that BMP7v selectively targets the CD44v6^+^ CSC compartment by counteracting its Wnt pathway activity and antiapoptotic machinery.

### BMP7v hampers the self-renewal capacity of CR-CSCs

**Fig. 3:**
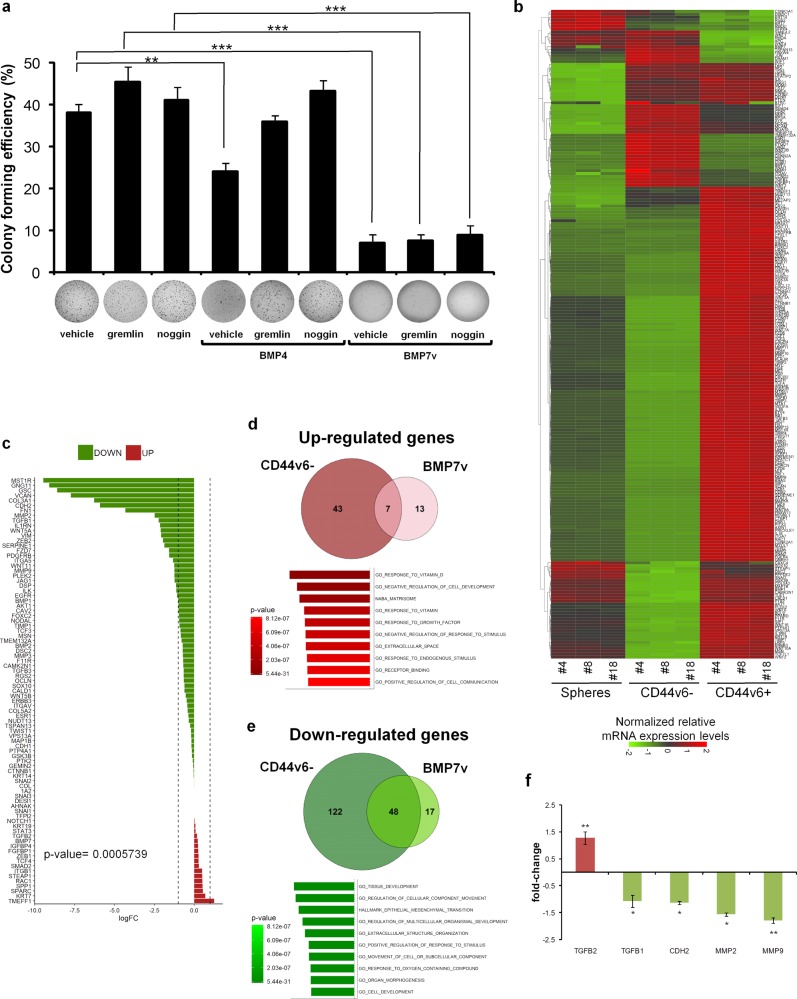
BMP7v hampers the self-renewal and recapitulates a CD44v6^−^-like cell subpopulation profile. **a** Colony forming efficiency percentage of CD44v6^+^ CR-CSCs treated for 14 days with vehicle, gremlin or noggin alone or in combination with BMP4 or BMP7v. Data are reported as mean ± SD of five different CRC sphere cell lines analyzed (CSC#1, 2, 4, 7, and 10). Representative soft-agar analysis is shown in the lower part of graph. **b** Heat map of EMT-, tumor metastasis-, and Wnt pathway-related genes (2^−ΔΔCt^ expression values) in spheres, CD44v6^−^ and CD44v6^+^ cells. Data are presented as normalized expression values of three different CRC sphere cell lines (CSC#4, 8, and 18). **c** Log fold change (logFC) values of differentially expressed related genes in enriched CD44v6^+^ cells treated with BMP7v for 3 days. Data are presented as the average of normalized mRNA expression levels of four different CR-CSC lines (CSC#1, 3, 5, and 7). Dotted lines represent −1 and 1 logFC values. *P* value indicates difference between normalized mRNA expression levels of untreated vs BMP7v treated samples. **d** Venn diagram showing upregulated (red) and **e** downregulated (green) genes in CD44v6^−^ and BMP7v treated cells. (Lower panels) Top ten significantly enriched gene sets (FDR *q* value ≤ 0.05), selected by using Hallmark, KEGG, and GO, related to the indicated 7 up- and 48 down-regulated genes common in CD44v6^−^ cells and CD44v6^+^ cells treated with BMP7v. *P* values related to each enriched gene set are indicated. **f** Fold change values of the differentially upregulated (red) and downregulated (green) genes further validated by RT-PCR in CR-CSCs upon treatment with BMP7v for 72 h. Data are expressed as mean ± SD of experiments performed in CSC# 1, 3, 9, and 21

We have already reported that BMP7v activity is resistant to the majority of BMP antagonists, such as noggin and chordin [31]. Given that only some cancers express high levels of gremlin and noggin [39, 40] we sought to investigate their inhibitory effects on both BMP4 and BMP7v. Although BMP4 was not able to accomplish its function in the presence of gremlin and noggin, BMP7v reduced the colony forming capacity of CR-CSCs more efficiently than BMP4, even in the presence of BMP inhibitors (Fig. 3a). We have previously demonstrated that CD44v6^+^ cells show upregulation of PI3K activity and EMT-related genes [8]. Differentially expressed EMT-, tumor metastasis- and Wnt signaling-related genes in CD44v6^−^ cell compartments (Fig. 3b) were comparable with those in CD44v6^+^ CRC cells treated with BMP7v (Fig. 3c), in line with the ability of this compound to turn CD44v6^+^ CSCs into CD44v6^−^ differentiated cells. Specifically, BMP7v induced the upregulation of 7 genes and the downregulation of other 48 genes in both CD44v6^−^ cells and CD44v6^+^ cell fractions. Of note, the gene set enrichment analysis (GSEA) performed with the molecular signatures database (MSigDB) revealed the activation of programs associated with differentiation and attenuation of EMT and metastatic biological processes (Fig. 3d, e and Supplementary Fig. 4a, b). The most relevant downregulated and upregulated genes were further validated by RT-PCR (Fig. 3f). Thus, BMP7v forces CD44v6^+^ CR-CSCs towards a more differentiated phenotype.

### BMP7v potentiates the effects of standard therapy in naive and chemoresistant CR-CSCs

**Fig. 4:**
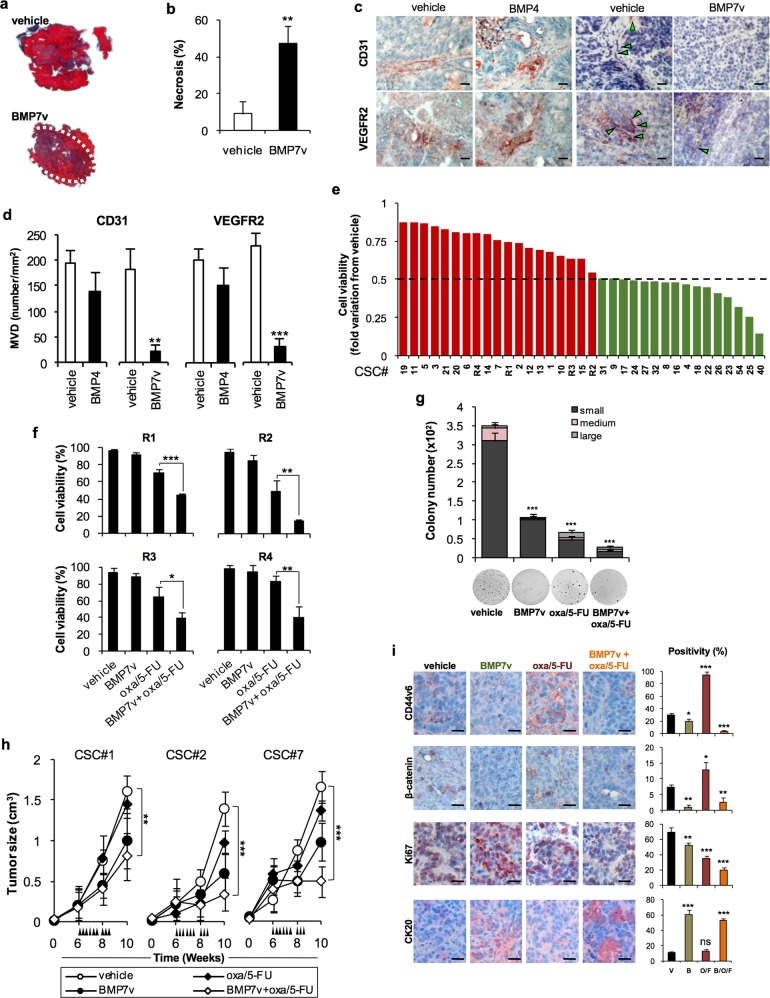
BMP7v exerts antiangiogenic effects and sensitizes chemoresistant CSCs to standard therapy. **a** Azan-Mallory staining on paraffin-embedded sections of xenografts derived from the injection of CRC sphere cells and treated for 4 weeks (6–9 weeks) with PBS (vehicle) or BMP7v. Data are representative of three independent experiments using different CRC sphere cell lines (CSC#2, 7, and 18). **b** Percentage of necrosis evaluated on paraffin-embedded sections of xenografts treated as in **a**. Data are shown as mean ± SD of three independent experiments. **c** Immunohistochemical analysis of CD31 and VEGFR2 (red staining) on paraffin-embedded sections of xenografts generated by the injection of CRC sphere cell lines and treated with PBS (vehicle), BMP4, or BMP7v. Green arrowheads indicate microvessels expressing CD31 or VEGFR2. Images are representative of three independent experiments using cells as in **a**. Nuclei were revealed by hematoxylin staining (blue). The scale bar represents 20 µm. **d** Number of microvessels positive for CD31 (left panel) and VEGFR2 (right panel) expression, evaluated on paraffin-embedded sections of xenografts treated as in **c**. Data are shown as mean ± SD of cells. MVD = microvessel density. **e** Fold change of viable cells in 35 CR-CSC lines treated with oxaliplatin/5-FU for 24 h. Dotted line indicates the threshold between chemoresistant (red) and sensitive CR-CSCs (green). **f** Cell viability percentage in chemoresistant CR-CSCs (R1-R4) pretreated with BMP7 for 3 days and with oxaliplatin/5-FU (oxa/5-FU) for additional 24 h as indicated. Data are shown as mean ± SD of three different experiments performed in the indicated R-CSCs. **g** Colony forming efficiency of CR-CSCs treated as in **f** and evaluated at 21 days. Representative soft-agar analyses are reported in the lower part of the graph. Bars show the mean ± SD of seven different CRC sphere cell lines (CSC#1–3, 5, 7, 10, and 18). **h** Tumor size of subcutaneous growth of the indicated CR-CSCs. Mice were treated for 4 weeks (6–9 weeks) with vehicle, oxaliplatin/5-FU (oxa/5-FU) and BMP7v alone or in combination. Error bars show the mean ± SD of tumor size measured in six mice/group. Black arrowheads indicate days of treatment. **i** Immunohistochemical analysis of CD44v6, β-catenin, Ki67, and CK20 (red color) in paraffin-embedded sections of CSC#7 xenografts treated as in **h**. Nuclei were counterstained by aqueous hematoxylin (blue color). The scale bar represents 20 µm (left panels). Percentage of CD44v6, β-catenin, Ki67, and CK20 positive cells in paraffin-embedded sections of tumor xenografts treated with vehicle (V), BMP7v (B), oxaliplatin/5-FU (O/F), alone or in combination (B/O/F) for 72 h. Error bars are mean ± SD of positive cell counts in three serial embedded-paraffin sections of six tumor xenografts per group derived from the injection of three different CRC sphere cells (CSC#1, 2, and 7) (right panels)

Following a dose-escalation delivery in vivo, we selected for subsequent studies the 50 μg/kg dose of BMP7v, which reduced significantly tumor growth while being well tolerated (Supplementary Fig. 5a). Of note, BMP7v alone exerted an antiangiogenic effect in xenograft tumors generated by the injection of CR-CSCs. Unlike BMP4, BMP7v treatment induced a fivefold increase in necrosis (Fig. 4a, b) and significantly reduced the number of microvessels in CR-CSC-based mouse avatars, as assessed by CD31 and VEGFR2 detection (Fig. 4c, d). In order to test whether BMP7v could sensitize CR-CSCs resistant to conventional therapy, we investigated the in vitro effect of BMP7v in combination with a standard chemotherapy regimen.

About half of the examined CR-CSC lines showed >50% survival after 24 h of in vitro treatment with oxa plus 5-FU (Fig. 4e). Based on the functional characterization of chemotherapy response, we selected four primary CR-CSC lines, derived from metastatic liver lesions of patients progressing after chemotherapy treatment (R1-R4), and three CR-CSC lines (CSC#1, 2, and 7) showing different degrees of chemoresistance. Of note, BMP7v was able to render R-CSCs sensitive to oxa plus 5-FU in vitro treatment, the same therapeutic regimen completely ineffective in vivo in the metastatic patients from which these cells were derived (Fig. 4f).

The ability to form colonies was significantly compromised in CR-CSCs in the presence of BMP7v in combination with oxa plus 5-FU in vitro (Fig. 4g). This treatment affected both the in vitro self-renewal and the in vivo tumorigenic capacity of subcutaneously injected CR-CSCs. While tumor xenografts generated by the injection of CSC#2 and 7 were sensitive to the combined treatment of BMP7v and chemotherapy, *PIK3CA*-mutant tumor xenografts derived from the implantation of CSC#1 delayed the outgrowth showing a kinetic trend similar to that of tumors treated with vehicle (Fig. 4h). Immunohistochemical analysis of xenograft tumors (CSC#7) treated with BMP7v and chemotherapy showed a reduction of CD44v6, nuclear β-catenin, and Ki67 expressing cells, together with a concomitant increase in CK20 positive cells (Fig. 4i). Interestingly, xenograft tumors treated with chemotherapy alone showed a significant increase of cells expressing CD44v6 and nuclear β-catenin accompanied with a decrease of Ki67 positive cells. Thus, BMP7v sensitizes both naive and chemoresistant CR-CSCs to standard therapies.

### BMP7v enhances the therapeutic response to PI3K inhibitors and reduces the size of PIK3CA-mutant xenograft tumors

**Fig. 5:**
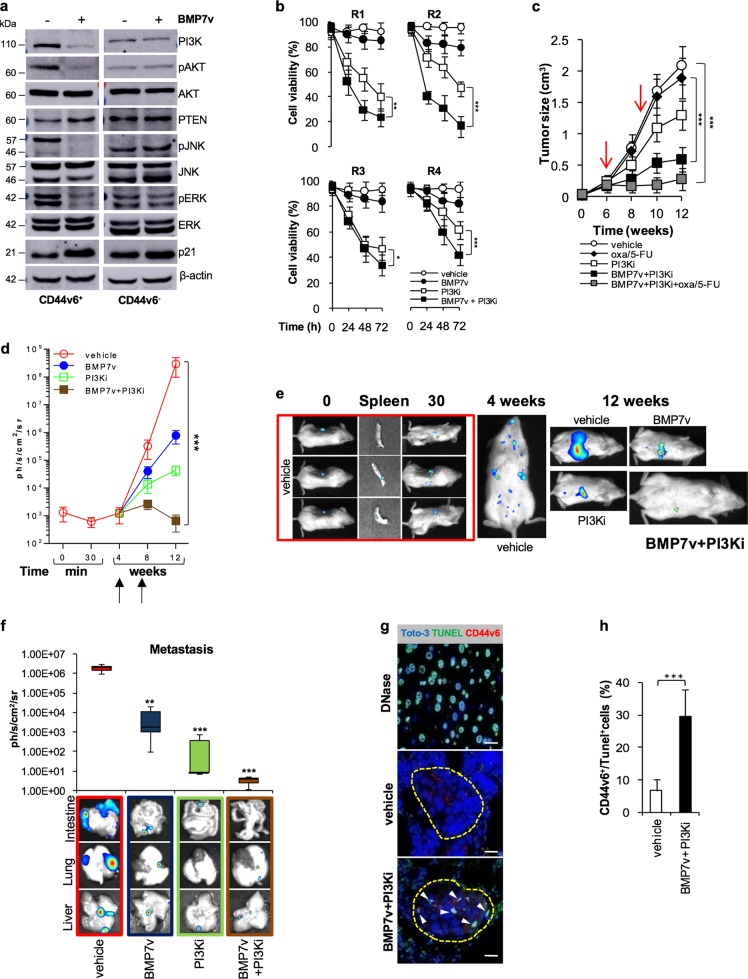
BMP7v in combination with PI3K inhibitor hampers tumor growth and reduces the metastatic lesion size. **a** Immunoblot analysis of PI3K, pAKT, AKT, PTEN, pJNK, JNK, pERK, ERK, and p21 in CD44v6^+^ and CD44v6^−^ cells treated with vehicle or BMP7v for 3 days. β-actin was used as loading control. One representative of three independent experiments (CSC#1, 4, and 7) is shown. **b** Cell viability percentage in R-CSCs treated with vehicle, BMP7v, PI3K inhibitor (PI3Ki), or BMP7v in combination with PI3K inhibitor (BMP7v + PI3Ki) up to 72 h. Data are shown as mean ± SD of three different experiments performed with the indicated R-CSCs. **c** Tumor size of subcutaneous outgrowth of *PIK3CA*-mutated xenografts. Mice were treated with vehicle, PI3K inhibitor (PI3Ki), oxaliplatin/5-FU (oxa/5-FU), BMP7v in combination with PI3K inhibitor (BMP7v + PI3Ki) or BMP7 in combination with PI3K inhibitor and oxaliplatin/5-FU (BMP7v + PI3Ki + oxa/5-FU). Data are shown as mean ± SD of tumor size of six mice/group using CSC#1, 18, and 25. Red arrows indicate the start and the end (from 6 to 9 weeks) of treatments. **d** Kinetics of metastasis formation detected by in vivo imaging analysis at the indicated time following spleen injection of CSC#1, 18, and 25 treated with vehicle, BMP7v, PI3K inhibitor (PI3Ki), or BMP7v in combination with PI3K inhibitor (BMP7v + PI3Ki) for 4 weeks. Black arrows indicate the start and end of treatments (from 4 to 7 weeks). Data are expressed as mean ± SD of six mice analyzed. **e** In vivo whole-body imaging analysis of mice treated as in **d** and analyzed at the indicated time points after splenectomy. **f** Photons count of all metastatic sites (liver, lung, and intestine) in mice treated as in **d**. Error bars are reported as mean ± SD of the xenografts analyzed as in **d** (upper panel). Representative in vivo imaging analysis of metastatic foci in the liver, lung, and intestine of mice treated as indicated (lower panels). **g** Immunofluorescence analysis of CD44v6 (red color) and TUNEL (green color) in paraffin-embedded sections of lung metastasis generated by the injection of CSC#25 in mice treated with vehicle or BMP7v + PI3K inhibitor (BMP7v + PI3Ki). White arrowheads indicate CD44v6^+^/Tunel^+^ CRC cells. Nuclei were counterstained with Toto-3 (blue color). Positive control was performed treating cells with DNase. The scale bars represent 20 µm. **h** Percentage of CD44v6^+^/Tunel^+^ cells of lung metastasis treated with vehicle or BMP7v + PI3K inhibitor (BMP7v + PI3Ki). Data are mean ± SD of xenografts derived from injection of three different cell lines (CSC#1, 18, and 25)

**Fig. 6:**
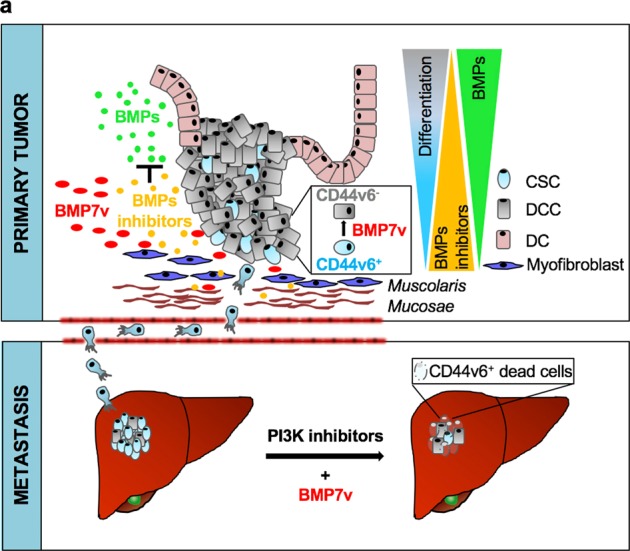
Schematic model of BMP7v effects in primary and metastatic CRC. **a** In primary tumor the colon cancer crypt organization, which is physiologically maintained by BMPs/BMP inhibitor balance, is disrupted. The administration of BMP7v selectively counteracts the expansion of the CSC compartment by reducing CD44v6 expression. Moreover, BMP inhibitors (noggin, gremlin, and others), produced by myofibroblasts, are not able to inhibit BMP7v activity on promoting the differentiation of CSCs (upper panel). In metastatic tumor, BMP7v in combination with PI3K inhibitors reduces the number of CD44v6^+^ cells and hampers the tumor metastatic growth (bottom panel). CSC cancer stem cell, DCC differentiated cancer cell, DC differentiated cell

We next investigated whether the addition of PI3K inhibitors to BMP7v-based therapy could represent an efficacious approach in the CD44v6^+^ cells, particularly in the presence of *PIK3CA* mutation. We first observed that in vitro BMP7v treatment attenuated the PI3K/AKT pathway in CD44v6^+^ cells, whose expression levels became similar to those exhibited by CD44v6^−^ compartment (Fig. 5a and Supplementary Fig. 5b). Then we found that BMP7v combined with a PI3K inhibitor (taselisib) was also able to revert the intrinsic chemotherapy resistance of CR-CSCs in vitro (Fig. 5b). Following the integration of the dose-response and synergy score evaluation from Bliss and ZIP algorithms, 100 ng/ml of BMP7v and 1 µM of PI3K inhibitor (taselisib) were selected for in vitro therapeutic combination (Supplementary Fig. 5c). Thus, to render *PIK3CA*-mutant CR-CSCs-derived avatars more sensitive to the combination therapy, we decided to inhibit the PI3K activity concomitantly. As expected, BMP7v in combination with taselisib significantly reduced the size of tumor xenografts generated by the injection of *PIK3CA*-mutated CR-CSCs (Fig. 5c), suggesting the necessity to simultaneously add a PI3K inhibitor in the presence of enhanced activation of the PI3K/AKT pathway. To determine whether the addition of BMP7v to PI3K inhibitor, taselisib could also induce regression of the disease, metastatic mouse avatars generated by the injection of *PIK3CA*-mutated CR-CSCs into NOD-SCID mice spleen, were treated 4 weeks after the splenectomy once the metastatic lesions were detectable. The combination of PI3K and BMP7v significantly lessened the size of metastatic lesions of *PIK3CA-*mutated CR-CSCs, even 4 weeks after treatment suspension (Fig. 5d-f). The majority of CD44v6^+^ cells found in the liver and lung metastases of mice treated with the PI3Ki-BMP7v combination therapy underwent cell death, confirming the potential clinical application of this therapeutic approach in patients with metastatic CRC (Fig. 5g, h). Altogether, these findings suggest that BMP7v can turn CD44v6^+^ cells into a therapy sensitive CD44v6^−^ differentiated phenotype. Although *PIK3CA*-mutated CR-CSCs are less sensitive to the prodifferentiation activity of BMP7 signaling, the addition of a PI3K inhibitor restores their sensitivity in established tumors (Fig. 6a).

## Discussion

We previously demonstrated that CD44v6 is a functional receptor that identifies migrating CSCs able to develop CRC metastasis [8]. Here, we show that CD44v6 enriched CR-CSCs lack the expression of BMP7, which is conversely confined within the differentiated counterpart (CD44v6^−^ cells). The heterogeneous expression of BMP7 within the CD44v6^−^ compartment is likely due to the presence of progenitor cells (CD44v6^−^/CD133^+^ cells) endowed with residual β-catenin activation, which renders these cells reprogrammable by the microenvironmental cytokines [8]. Here, we show that BMP7 expression represents an early event in CRC as confirmed by its presence in colon adenoma and adenocarcinoma. Moreover, its expression inversely correlated with the pathological grading of CRCs, again supporting the association between this morphogen and differentiated tumor. In accordance with the reported effects in glioma stem cells [41], BMP7v induce the expression of differentiation markers in CR-CSCs, which gradually reduce their β-catenin activity and CD44v6 expression. We previously reported that BMP4 promotes differentiation and affects the self-renewal activity of CR-CSCs [24]. Through a *SMAD1/4*-mediated epigenetic mechanism, BMP4 leads to the recruitment of histone deacetylase HDAC1 and consequent transcriptional suppression of stemness genes [42]. Accordingly, targeting the Wnt pathway by BMP7v curtails the clonogenic activity of CD44v6^+^ CRC cells and leads to their terminal differentiation, as highlighted by the presence of PARP activation and downregulation of the antiapoptotic proteins Bcl-2 and Bcl-xL. The similar trend of cell viability in both CD44v6^+^ and CD44v6^−^ populations is likely due to the presence, in the CD44v6^−^ fraction, of progenitor cells that are affected by the BMP7v treatment. Tumor microenvironment reprograms cancer cells towards an EMT process leading them to acquire a more pronounced self-renewal and migratory phenotype [8, 43]. We have shown that cytokines released by cancer-associated fibroblasts are able to dedifferentiate CD44v6^−^ cells into CD44v6^+^ metastatic CR-CSCs, with the induction of a number of EMT genes [8]. BMP7v drives CR-CSCs to behave similarly to the differentiated CD44v6^−^ cells by downregulating the majority of EMT-related genes, which are needed by CD44v6^+^ cells to retain an aggressive phenotype. We previously demonstrated that BMP4 induces PTEN upregulation and inhibition of PI3K/AKT [24], which sustains the stem-like cell properties of CD44v6^+^ CR-CSCs [8]. BMP7v displays a similar inhibition on the PI3K/AKT pathway. Here, we have shown that even though PTEN increased moderately upon BMP7v treatment, PI3K protein expression, and AKT activation levels decreased significantly in CD44v6^+^ cells, which differentiate and revert to CD44v6^−^/PI3K^low^ cells. This mechanism is in line with the observation that the natural compound resveratrol inhibits the PI3K pathway by upregulating the BMP7 in human colon cancer cells [44]. The induction of differentiation may represent an alternative therapeutic approach to render CSCs sensitive to standard therapies [24, 45]. Differentiation therapy is currently in phase 2 clinical trial for the cure of acute promyelocytic leukemia [46]. The expression of antiapoptotic genes and the upregulation of survival factors contribute to CSC resistance to conventional anticancer therapies [32].

BMP7v reduces the expression levels of anti-apoptotic proteins and makes *PIK3CA* wt CRC avatars sensitive to standard chemotherapy. In addition, BMP7v enhances the therapeutic response even against CR-CSCs purified from metastatic lesions of patients who underwent chemotherapy. About 10–30% of CRCs exhibit PI3K activation sustained by *PIK3CA* mutations, which contribute to confer resistance to standard therapies and targeted agents [47]. *PIK3CA*-mutated tumor xenografts display resistance to the combination of BMP7v and chemotherapy. This is not surprising because it is likely that the increased β-catenin activation promoted by the constitutive activation of PI3K counteracts the inhibitory activity on the Wnt pathway induced by BMP signaling.

PI3K is a target for pharmacological drug design and therapeutic intervention in many cancers, including CRC [48]. Nowadays, although PI3K inhibitors have a limited effect as single agents in CRC, several clinical trials are exploring the efficacy of these inhibitors in different combinatorial treatments [47, 49]. Here we showed that BMP7v is able to sensitize chemoresistant CR-CSCs to PI3K inhibitors in vitro regardless their MSI and CMS profiles. Addition of a PI3K inhibitor to the combination of BMP7v and chemotherapy-induced tumor regression of *PIK3CA*-mutant tumor xenograft, further supporting the potential clinical application of this combination therapy. In line with our previous findings, PI3K inhibitors alone selectively target disseminating CR-CSCs [8], whereas in combination with BMP7v treatment induce disease regression by reducing the size of both primary tumors and established CRC liver metastasis.

In conclusion, we have shown that BMP7v exerts a potent antitumor activity through the induction of differentiation of *PIK3CA* wt CR-CSCs. Although the presence of *PIK3CA* mutation reduces the therapeutic activity of BMP7v, we provide evidence that the addition of PI3K inhibitors may be sufficient to restore CR-CSC sensitivity to BMP7v. Unlike BMP4, BMP7v exerts a significant antiangiogenic effect and can be administered systemically, due to its solubility and prolonged half-life, which also facilitate its combination with standard chemotherapy or targeted agents. Further studies are needed to investigate the minimal residual disease upon the combination therapy based on BMP7v and PI3K inhibitor.

Because the efficacy of targeted therapy is limited by the presence of intratumor heterogeneity, this prodifferentiation approach coupled with such a considerable antiangiogenic activity may contribute to overcome the hurdles of dynamic tumor changes. Thus, BMP7v-based combination therapies may represent potential novel treatment options for CRC.

## Materials and methods

### Isolation and treatment of CR-CSCs

Human CRC tissues were obtained from 40 patients at the time of resection, in accordance with the ethical standards of the Institutional Committee on Human Experimentation (authorization CE9/2015, Policlinico Paolo Giaccone, Palermo) after informed consent. Peritumoral mucosa was recovered from the uninvolved surrounding tumor tissue. Clinical data of CRC patients from which CRC sphere cell lines were derived are reported in Supplementary Table 1. CRC sphere cells were isolated and propagated as previously described [8, 32]. Briefly, specimens were digested in DMEM medium supplemented with 10 µg/ml of hyaluronidase (Sigma) and 0.6 mg/ml of collagenase (Gibco) for 1 h at 37 °C, resuspended in serum-free stem cell medium comprising EGF (Peprotech) and FGF2 (Peprotech) and cultured in ultra-low adhesion flasks. To generate SDACs, CR-CSCs were dissociated and then cultured in DMEM-high glucose supplemented with 10% FBS in adherent conditions up to 21 days [24]. Gradual morphological differentiation was determined by counting of cells in adherent conditions normalized with the number of CR-CSCs in suspension. To evaluate differentiation of CRC organoids, CR-CSCs were dissolved in a 1:10 stem cell medium/Matrigel solution, placed in a 24-well plate as a single drop covered by medium and monitored twice a week up to 21 days.

Authentication of CRC sphere cell lines was assessed by short tandem repeat (STR) DNA profiling (GlobalFiler™ STR kit, Applied Biosystem) using the ABIPRISM 3130 genetic analyzer (Applied Biosystem) as recommended by the manufacturer’s instructions. STR profiles of CRC sphere cells were matched with their relative patient-derived tumors. Primary cultures enriched in CSCs derived from chemoresistant metastatic liver lesions were obtained from patients undergoing hepatectomy with curative intent at the University Polyclinic A. Gemelli, Rome. Cell cultures were monitored for the presence of mycoplasma. CRC sphere cells and their enriched CD44v6^+^ and CD44v6^−^ fractions were treated with BMP7v (100 ng/ml produced by Eli Lilly as previously described) [30], gremlin (1 µg/ml, R&D Systems), noggin (100 ng/ml, R&D Systems), BMP4 (100 ng/ml, R&D Systems), oxa (10 µM, Selleckchem), 5-FU (10 µM, Selleckchem), and taselisib (PI3Ki, 1 µM GDC-0032, Chemietek). Oxa was added to cell culture media 2 h before 5-FU treatment. All the compounds above described were added to cell culture media every 48 h. BMP7v dose was determined by the evaluation of colony forming efficiency in presence of different concentration of BMP inhibitors (gremlin and noggin), when in combination with PI3K inhibitor (taselisib) its effective dose was selected on the basis of CR-CSC viability. These experiments were conducted in SynergyFinder, including Bliss and ZIP, to calculate dose-response inhibition matrix and synergy scores [50].

### Immunohistochemistry and Immunofluorescence

Immunohistochemistry analysis was performed on 5-μm-thick paraffin-embedded sections of CRC tissues or CRC sphere cell-derived tumor xenografts. For intracellular epitope detection, tissue sections were permeabilized in ice-cold 0.1% TritonX-100 in PBS for 10 min. Tissue samples were exposed overnight at 4 °C to specific antibodies for BMP7 (MAB3541, mouse, IgG2_b_, R&D system), E-cadherin (#3195, rabbit, IgG, CST), CD31 (M0823, clone JC70A, mouse IgG1_k_, Dako), VEGFR2 (AF357, goat, IgG, R&D System), CD44v6 (BBA13, clone 2F10, mouse, IgG1, R&D system), β-catenin (sc-7199, rabbit, IgG, Santa Cruz Biotechnology), Ki67 (M7240, mouse, IgG1, DakoCytomation), and CK20 (NCL-L-CK20, mouse, IgG2_k_, Novocastra Leica). Primary antibodies were revealed by biotin-streptavidin peroxidase LSAB 2 Kit (Dako). Stainings were detected by using 3-amino-9- ethylcarbazole chromogen. Nuclei were counterstained with aqueous hematoxylin (Sigma).

For Azan-Mallory, tissues were stained with azocarmine G (Sigma) and 5% of phosphoric acid. Then, sections were stained with a Mallory mix solution (Sigma). Staining was analyzed by using Imaging Analyzer Software.

Immunofluorescence staining was performed on paraffin-embedded sections, cells cytospun or cultured on coverslips. Cells were fixed in 2% paraformaldehyde for 20 min at 37 °C. Intracellular epitope detection was performed in cells permeabilized in 0.1% TritonX-100 in PBS for 10 min. Following blocking with 3% bovine serum albumin (BSA) for 30 min, cells were exposed overnight at 4 °C to BMP7 (MAB3541, mouse, IgG2_b_, R&D system), LGR5 (GPR49, rabbit, IgG, Abgent), CDX2 (MAB3665, mouse, IgG1, R&D Systems), CK20 (NCL-L-CK20, mouse, IgG2_k_, Novocastra Leica), E-cadherin (#3195, rabbit, IgG, CST), vimentin (#5741, rabbit, IgG, CST), β-catenin (MAB1329, mouse, IgG2_b_, R&D Systems), CD44v6 (BBA13, clone 2F10, mouse, IgG1, R&D system), BMPR1A (MAB2406, mouse, IgG2_b_, R&D Systems), BMPR1B (MAB505, mouse, IgG2_a_, R&D Systems) and, BMPR2 (MAB811, mouse, IgG2_b_, R&D Systems) antibodies or isotype-matched controls (IMCs). Primary antibodies were revealed using Alexa Fluor 488 or Rhodamine-conjugated anti-mouse or anti-rabbit secondary antibodies (Invitrogen) in the presence of RNase (40 μg/ml, Sigma). Nuclei were counterstained using Toto-3 iodide (Molecular Probes).

Cell quantitation was performed by ImageJ Software analysis. Tunel assay was performed by using In Situ Cell Death Detection, AP Kit(Roche Diagnostics GmbH). DNA strand breaks were detected by 5-bromo-4-chloro-3-indolyl-phosphate (, Dako) substrate. DNase was used to perform the positive control.

TMAs were provided by TriStar Technology group (*Tri*), LLC, 9700 Great Seneca Highway, Rockville, MD 20850.

### Flow cytometry and cell cycle analysis

CD133 and CD44v6 positive and negative subpopulations were obtained using FACS cell sorter (BD). Cells were stained for 1 h at 4 °C with CD133-PE (130-090-851, 293C3, mouse IgG2_b_, Miltenyi), CD44v6-APC (FAB3660A, clone 2F10, mouse, IgG1, R&D systems) or corresponding IMCs IgG2b-PE (#130-092-215, mouse, Miltenyi) or IgG1-APC (#IC002A, mouse, R&D systems). Before sorting, cells were resuspended in PBS supplemented with 2% BSA and 2 mM EDTA, and strained through a 70 µm mesh to avoid cell hindrance. Dead cells were excluded with 7-AAD (BD). Quality of postsorting was verified by flow cytometry using specific antibodies against CD133 (170-070-702, CD133/1-APC, AC133, mouse IgG1_k_, Miltenyi) or CD44v6 (#MA5-16966, CD44v6-FITC, VFF-7, mouse IgG1, Thermo Scientific). Cells were then washed twice in PBS, permeabilized with fixation/permeabilization solution (Cytofix/Cytoperm, BD) following the manufacturer’s instructions and stained with BMP7-FITC (FCMAB135F, 2A10, mouse IgG1_k_, Merck) or its IMC IgG1_k_ (#F6397, mouse, Sigma-Aldrich). For flow cytometry analysis of CD133 and CD44v6, PE- (293C3) or APC-conjugated (2F10) clone were used, respectively. Cell cycle analysis was performed by quantification of DNA content on dissociated cells. Cells were incubated overnight at 4 °C in a buffer containing 50 μg/ml propidium iodide (Sigma-Aldrich), 0.1% sodium citrate (Sigma-Aldrich), 0.1% TritonX-100 and 10 μg/ml RNAse (Sigma-Aldrich). All data were analyzed using FlowJo software (Tree Star). The percentage of CD44v6^+^ cells has been assessed in all the 35 CRC sphere cell lines and indicated as low (<30%), medium (30–70%), and high (>70%) in Supplementary Table 2.

### Lentiviral particles generation and CR-CSC transduction

To generate lentiviral particles, packaging cell line HEK-293T were transfected with second-generation packaging plasmids (PSPAX2 and pMD2.G plasmids Addgene) in association with p-TWEEN LUC or TOP-dGFP (Addgene # 35489) lentiviral vectors. Transfection was performed using X-tremeGENE HP DNA Transfection Reagent (Roche). Lentiviral supernatants were collected and concentrated with the Lenti-X Concentrator reagent (Clontech). 1 × 10^5^ CRC sphere cells were transduced with concentrated viral supernatants for 24 h using 8 μg/ml polybrene. Wnt pathway activity was monitored by flow cytometry analysis on the basis of the TOP-dGFP expression levels.

### Cell viability, clonogenic, and invasion assay

The cell viability assay was performed using the CellTiter96^®^ Aqueous One Solution Cell Proliferation Assay Kit (Promega) according to the manufacturer’s instructions and examined with GDV programmable MPT reader (DV 990 BV6). For invasion assay, 2 × 10^3^ dissociated CR-CSCs were plated into 8 µm pore size matrigel (BD)-coated transwell and treated with vehicle or BMP7v up to 48 h. Supernatant of NIH-3T3 cells cultured in serum-free medium was used as chemoattractant in the lower part of the transwell system. Migrating cells were examined and counted using an optical microscope. For clonogenicity, CR-CSCs were plated at a clonal density on Agarose Sea Plague Agar (Invitrogen) and maintained up to 21 days. Colonies were stained with 0.01% Crystal Violet, evaluated based on their size (small 30–60 µm, medium 60–90 µm, and large >90 µm) and counted using ImageJ software.

### Western blot analysis

Cell pellets were resuspended in ice-cold lysis buffer (50 mM Tris-HCL pH 8, 150 mM NaCl, 0.5% sodium deoxycholate, 0.1% SDS, 1% NP40, 1 mM EDTA) supplemented with a mix of protease and phosphatase inhibitors (Thermo Fisher Scientific). Equal amount of protein extracts was resolved on SDS-PAGE gels and blotted on nitrocellulose membranes. Membranes were exposed overnight at 4 °C to PARP (#9524, rabbit, IgG, CST), Caspase-3 (#9662, rabbit, IgG, CST), cleaved Caspase-3 (Asp175) (#9661, rabbit, IgG, CST), Bcl-2 (sc-7382, mouse, IgG, Santa Cruz), Bcl-xL (sc-8392, mouse, IgG, Santa Cruz), PI3K (p110α) (#4249, C73F8, rabbit, IgG, CST), phospho-AKT (Ser473 XP) (#4060, D9E, rabbit, IgG, CST), AKT (#9272, rabbit, IgG, CST), PTEN (#9559, 138G6, rabbit, IgG, CST), phospho-JNK (Thr183/Tyr185) (#4668, 81E11, rabbit, IgG, CST), JNK (#9258, 56G8, rabbit, IgG, CST), phospho-ERK (sc-7383, E-4, mouse, IgG2_a_, Santa Cruz), ERK (sc-94, rabbit, IgG, Santa Cruz), p21 (#2946, DCS60, mouse IgG2a, CST), and β-actin (#3700, 8H10D10, mouse IgG2_b_, CST). Primary antibodies were detected using specific secondary HRP-conjugated antibodies (Thermo Fisher Scientific) and chemiluminescence signals were revealed using Amersham imager 600 (GE Healthcare). Protein expression levels were measured by densitometry analysis using ImageJ software.

### Mutation analysis and MSI profile

Total DNA was purified from CR-CSCs using the Blood and Tissue Kit (QIAGEN). The mutational status of *KRAS, BRAF, PIK3CA*, and *SMAD4* genes was evaluated by the BigDye Terminator v3.1 Cycle Sequencing Kit (Applied Biosystems) using the following primers specific for *KRAS*^G12/G13^ (F-ATCGTCAAGGCACTCTTGCCTAC, R-GTACTGGTGGAGTATTTGATAGTG), *BRAF*^V600^ (F-ACTCTAAGAGGAAAGATGAAG, R-GTGAATACTGGGAACTATGA), *PIK3CA*^E545^ (F-ATTGTTCACTACCATCCTC, R-TAATGTGCCAACTACCAATG) and *SMAD4*^R361^ (F-TGTGGAGTGCAAGTGAAAGC, R-TCAATGGCTTCTGTCCTGTG). Codon Code Aligner Software was used for sequence assembly and alignment. Assessment of MSI status of CR-CSCs was carried out using the GeneQuality CC-MSI kit (Analitica Advanced Biomedicine). Purified total DNA was subjected to a multiplex microsatellite PCR including mononucleotide repeats (BAT25, BAT26, BAT40, NR21, NR24, and TGFβRII) and dinucleotide repeats (D2S123, D17S250, D5S346, and D18S58). MSI analysis was carried out using GeneMapper 5.0 Software (Applied Biosystems). Samples were classified as MSI-high (four or more markers instable), MSI-low (1–3 markers instable), or MSS (microsatellite stable). Mutation and MSI profiles of CR-CSCs were performed on a Genetic Analyzer ABIPRISM 3130 (Applied Biosystems). COSMIC-reported mutations of *KRAS, BRAF, PIK3CA*, and *SMAD4* in the 35 CRC sphere cell lines used and their MSI profiles are indicated in Supplementary Table 2.

### RNA isolation and Real-time PCR

Total RNA was obtained using the RNeasy Plus Mini Kit (Qiagen GmbH) according to the manufacturer’s instructions. The yield of the extracted RNA was determined by Nanodrop ND-1000 (Nanodrop, Wilmington, DE). One microgram of total RNA was retro-transcribed using the High-Capacity cDNA Archive Kit (Applied Biosystems) following the standard protocol. Quantitative real-time PCR analysis was performed in SYBR Green PCR master mix (SuperArray Bioscience) containing primers for *BMPR1A* (F-GTCATACGAAGATATGCGTGAGGTTGT, R-ATGCTGTGAGTCTGGAGGCTGGATT), *BMPR1B* (F-AAGGCTCAGATTTTCAGTGTCGGGA, R-GGAGGCAGTGTAGGGTGTAGGTCTTTATT), *BMPR2* (F-GTGACTGGGTAAGCTCTTGCCGTCT, R-GCAGGTTTATAATGATCTCCTCGTGGT), *TGFβ1* (F- CTCGCCCTGTACAACAGCA, R-GGTTTCCACCATTAGCACGC), *TGFβ2* (ACAGACCCTACTTCAGAATTGTT, R-TGGGTTCTGCAAACGAAAGA) *CDH2* (F- GGAGAACCCCATTGACATTGT, R-TGTTCCAGGCTTTGATCCCT), *MMP2* (TGGTGGGAACTCAGAAGGTG, R-CCACATCTTTCCGTCACTGC), *MMP9* (F- ACTACTGTGCCTTTGAGTCC, R-CCAGTACTTCCCATCCTTGA), or *GAPDH* (F-GCTTCGCTCTCTGCTCCTCCTGT, R-TACGACCAAATCCGTTGACTCCG). Relative mRNA expression levels were normalized with the endogenous control (*GAPDH*) and calculated using the comparative Ct method 2^−ΔΔCt^. mRNA expression levels of EMT-, tumor metastasis-, and Wnt pathway-related genes were detected by RT^2^ profiler PCR array (PAHS-090, Qiagen) according to manufacturers’ instructions. Data were analyzed using the R version 3.5.0 and plotted by the pheatmap version 1.0.10 and VennDiagram 1.6.20, gtools 3.8.1, and ggplot2 3.0.0. GSEA was performed by selecting the Kyoto Encyclopedia of Genes and Genomes, Gene Ontology and Hallmarks within MSigDB version 6.2. Gene sets with a False Discovery Rate *q* value ≤ 0.05 were considered significantly enriched. CMS1–4 profile of CR-CSCs was based on the evaluation of RNA-seq data on matched specimens derived from primary lesions of CRC patients as reported in Linnekamp et al. [51]. Correlation analysis data were obtained using the “R2: Genomics Analysis and Visualization Platform” (http://r2.amc.nl http://r2platform.com) in CRC samples from the Expression Project for Oncology (GEO accession number GSE2109).

### Animals and tumor models

Dissociated CRC sphere cells (5 × 10^5^) were injected subcutaneously into the flank of 5–6-week-old male NOD-SCID mice (Charles River), in a total volume of 100 µl of serum-free medium mixed with matrigel (BD) in a ratio of 1:1. The Replacement, Reduction, and Refinement (3Rs) principles were used to estimate the lowest sample size (six mice per group).

Mice were treated for four weeks with PBS (vehicle) or BMP7v (50 μg/kg, 3 days/week) alone or in combination with oxa (0.25 mg/kg, once a week) and 5-FU (15 mg/kg, 2 days/week) by i.p. injection, and with taselisib (5 mg/kg, once daily) by oral gavage. Treatment with BMP4 was performed by intratumoral injection of 100 BMP4-coated beads once a week for 6 weeks. Heparin acrylic beads (Sigma) were incubated with BMP4 (0.65 μg/μl) for 1 h at 37 °C and washed twice in PBS.

Tumor size was calculated according to the formula: (*π*/6) × (smaller diameter)^2^ × larger diameter.

For in vivo migration experiments, 3 × 10^5^ luciferase (LUC)-transduced CD44v6^+^ CR-CSCs were resuspended in PBS and injected into the spleen of NOD/SCID mice. Following i.p. administration of d-luciferin (150 mg/kg, Promega), the bioluminescence signal of migrating cells was measured before and 30 min after the cell injection and immediately after splenectomy up to 12 weeks (every 4 weeks) by using Photon IMAGER instrument (Biospace). No randomization procedure was used. Investigators were not blinded during analysis.

### Statistical analysis

The sample size was chosen to reach a power of 0.9, 0.05 error probability and a large effect size (>0.5) for our groups of treatments. Data were presented as mean ± standard deviation. Statistical significance was estimated by Analysis of Variance (one way or two ways) with Bonferroni post test, or by unpaired *T* test. Results were referred to statistically significant as *P* < 0.05. * indicates *P* < 0.05, ** indicate *P* < 0.01, and *** indicate *P* < 0.001.

### Study approval

This study was performed in accordance with the ethical standards of the Institutional Committee on Human Experimentation (authorization CE9/2015, Policlinico Paolo Giaccone, Palermo). All animal experiments were approved by the Institutional Italian Guidelines for Animal Welfare of the University of Palermo (D.L. n° 26 March 4, 2014, Authorization #154/2017-PR; Protocol 2B952.5).

## Supplementary information

Supplementary Figure Legends

Supplementary Figure 1

Supplementary Figure 2

Supplementary Figure 3

Supplementary Figure 4

Supplementary Figure 5

Supplementary Table 1

Supplementary Table 2
