# Supplementary material for: Targeting chemoresistant colorectal cancer via systemic administration of a BMP7 variant
Source: Oncogene. 2019 Oct 7;39(5):987–1003. doi: 10.1038/s41388-019-1047-4 (PMC6989400; doi:10.1038/s41388-019-1047-4)
Supplement: Supplementary file 1 — Supplementary Figure Legends [file 41388_2019_1047_MOESM1_ESM.doc]

**Supplementary Figure Legends**

**Figure S1: CD44v6^+^ CR-CSCs lack BMP7 expression**

**a,** Correlation analysis between *BMP7* mRNA expression levels and the pathological grading in CRC samples from R2 database (Tumor Colon EXPO 350). P-value indicates that the negative correlation coefficient is statistically significant. **b,** Post-sorting analysis of CD133 in CD133 enriched cells. One representative of CSC#4, 8, 23-26 is shown. Grey histograms show the isotype matched controls (IMC). **c,** Percentage of BMP7 positive cells in enriched CD133^+^ and CD133^-^ CRC sphere cells as in (b) assessed by immunofluorescence. Data are expressed as mean ± SD of 6 different CRC sphere cell lines analyzed (CSC#4, 8, 23-26). **d,** CD44v6 expression on sorted CRC sphere cells as in (b) for CD44v6. **e,** Percentage of BMP7 positive cells evaluated by immunofluorescence analysis in CD44v6^+^ and CD44v6^-^ cells. Data are expressed as mean ± SD as in (c).

**Figure S2: BMP7v reduces the content of CD44v6^+^ CR-CSCs**

**a,** Relative mRNA expression levels of *BMPR1A*, *BMPR1B* and *BMPR2* in CD44v6^+^ and CD44v6^-^ cells. Data are expressed as mean ± SD of 5 different CRC sphere cell lines analyzed (CSC#1, 2, 4, 7, 10). **b,** Immunofluorescence analysis of BMPR1A, BMPR1B and BMPR2 (green color) in cells as in (a). Nuclei were counterstained with Toto-3 (blue color). The scale bars represent 20 µm. **c,** Dose-response curve of BMP7v treatment based on cell viability % at 24 hours. IC_50_ was calculated fitting a no linear regression algorithm. One representative experiment of CSC# 3, 7, 9, 21 is shown. **d,** Dose-response matrix indicating the inhibition percentage of CR-CSC colony forming efficiency following treatment with vehicle, gremlin (*left panel*) or noggin (*right panel*) alone or in combination with BMP7v for 14 days at the indicated dose concentrations. One representative experiment of CSC#1, 2, 7 is shown. **e,** Flow cytometry analysis of CD133 in CD44v6^+^cells treated with vehicle or BMP7v up to 14 days. One representative experiment of CSC#1, 2, 4, 7, 10 is shown. **f,** Fold MFI change (over time 0) of CD133 and CD44v6 expression in CD44v6^+^ CR-CSCs treated with BMP7v up to 14 days. Data are expressed as mean ± SD of 3 independent experiments performed in 5 different CRC sphere cell lines (CSC#1, 2, 4, 7, 10). **g,** Correlation analysis between *CDX2* mRNA expression levels and the pathological grading in CRC samples from R2 database (Tumor Colon EXPO 350). **h,** Fold MFI change (over time 0) of β-catenin activity (TOP-GFP) in CD44v6^+^ CR-CSCs treated with BMP7v up to 14 days. Data are expressed as mean ± SD of 3 independent experiments performed as in (f). **i,** Flow cytometry analysis of CD44v6^+^ CR-CSCs transduced with TOP-GFP and treated with vehicle (green histogram) or BMP7v (red histogram) for 14 days. Negative control is indicated by the grey histogram. One representative experiment of CSC#8, 9, 11 is shown.

**Figure S3: BMP7 expression levels are positively correlated with E-cadherin whereas inversely associated with pathological grading**

**a,** Correlation analysis between *CDH1* (E-cadherin) and *BMP7* mRNA expression levels in CRC samples from TCGA-COAD and TCGA-READ. **b,** Immunohistochemical analysis of E-cadherin (red staining) in xenograft tumors derived from injection of CR-CSCs treated with vehicle or BMP7v for 4 weeks. Two representative experiments of CSC# 1, 8, 25 are shown. Nuclei were counterstained by aqueous hematoxylin (blue color). The scale bar represents 100 µm *(left panels).* Box plot indicating the distribution of E-cadherin positive cell percentage measured in xenograft tumors treated as indicated in (b) *left panels*. Data are mean ± SD of 3 different CRC sphere cell lines (CSC#1, 8, 25) *(right panel).* **c,** Representative flow cytometry analysis of cell cycle in CD44v6^+^ CR-CSCs (CSC#2) treated with vehicle or BMP7v for 72 hours. Plots show the percentage of cells in sub-G0 phase (red box), G0-G1 phase (light yellow box), S phase (pink box) and G2-M phase (brown box). **d** and **e,** Optical density ratio of PARP, c-PARP, Caspase-3 (Casp-3), cleaved Caspase-3 (cCasp-3), Bcl-2, Bcl-xL expression levels in CD44v6^+^ and CD44v6^-^ cells treated with vehicle or BMP7v for 72 hours. Data are mean ± SD of 3 different CRC sphere cell lines (CSC#1, 4, 7).

**Figure S4: BMP7v turns CD44v6^+^ into CD44v6^-^-like gene expression profile**

**a,** Up- and **b** down-regulated genes and their relative involved gene sets, common in CD44v6^-^ cells and CD44v6^+^ cells treated with BMP7v, selected from Hallmark, KEGG and GO.

**Figure S5: BMP7v potentiates the effects of chemotherapy and attenuates the activity of PI3K/AKT pathway in CR-CSCs**

**a,** Tumor size of subcutaneous growth of CRC sphere cells treated for 4 weeks with vehicle or dose escalation of BMP7v. Arrows indicate the start and the end of treatment (from 6 to 9 weeks). Data represent mean ± SD of tumor size of 6 tumors per group using 3 different sphere cell lines (CSC#2, 7, 18). **b,** Relative band densities of PI3K, pAKT, AKT, PTEN, pJNK, JNK, pERK, ERK and p21 in CD44v6^+^ (*left panel*) and CD44v6^-^ (*right panel*) cells treated with vehicle or BMP7v for 72 hours. Data are expressed as mean ± SD of 3 independent experiments using different CRC sphere cell lines (CSC#1, 4, 7). **c,** Zip synergy score evaluation of BMP7v treatment alone or in combination with PI3K inhibitor (taselisib) in CR-CSCs for 24 hours at the indicated doses. Red area indicates doses of BMP7v and taselisib with synergistic effect. Dotted line indicates the highest synergistic area. One representative experiment of CSC# 3, 7, 9, 21 is shown.

**Supplementary Table 1:** Clinical data of CRC patients from which CRC sphere cell lines were derived.

**Supplementary Table 2: COSMIC-reported mutations of the indicated genes and MSI profile, CD44v6 expression, CMS in CR-CSC lines**

Bluish color indicates wild type genes in specific cell lines while blue, red, green and purple colors indicate KRAS, BRAF, PIK3CA and SMAD4 mutations, respectively. Percentage of mutation rate in CRC patients reported in COSMIC database (white bars) and in CRC sphere cell lines (black bars) is shown on the right.
