# Supplementary figures and images for: Targeting chemoresistant colorectal cancer via systemic administration of a BMP7 variant
Source: Oncogene. 2019 Oct 7;39(5):987–1003. doi: 10.1038/s41388-019-1047-4 (PMC6989400; doi:10.1038/s41388-019-1047-4)

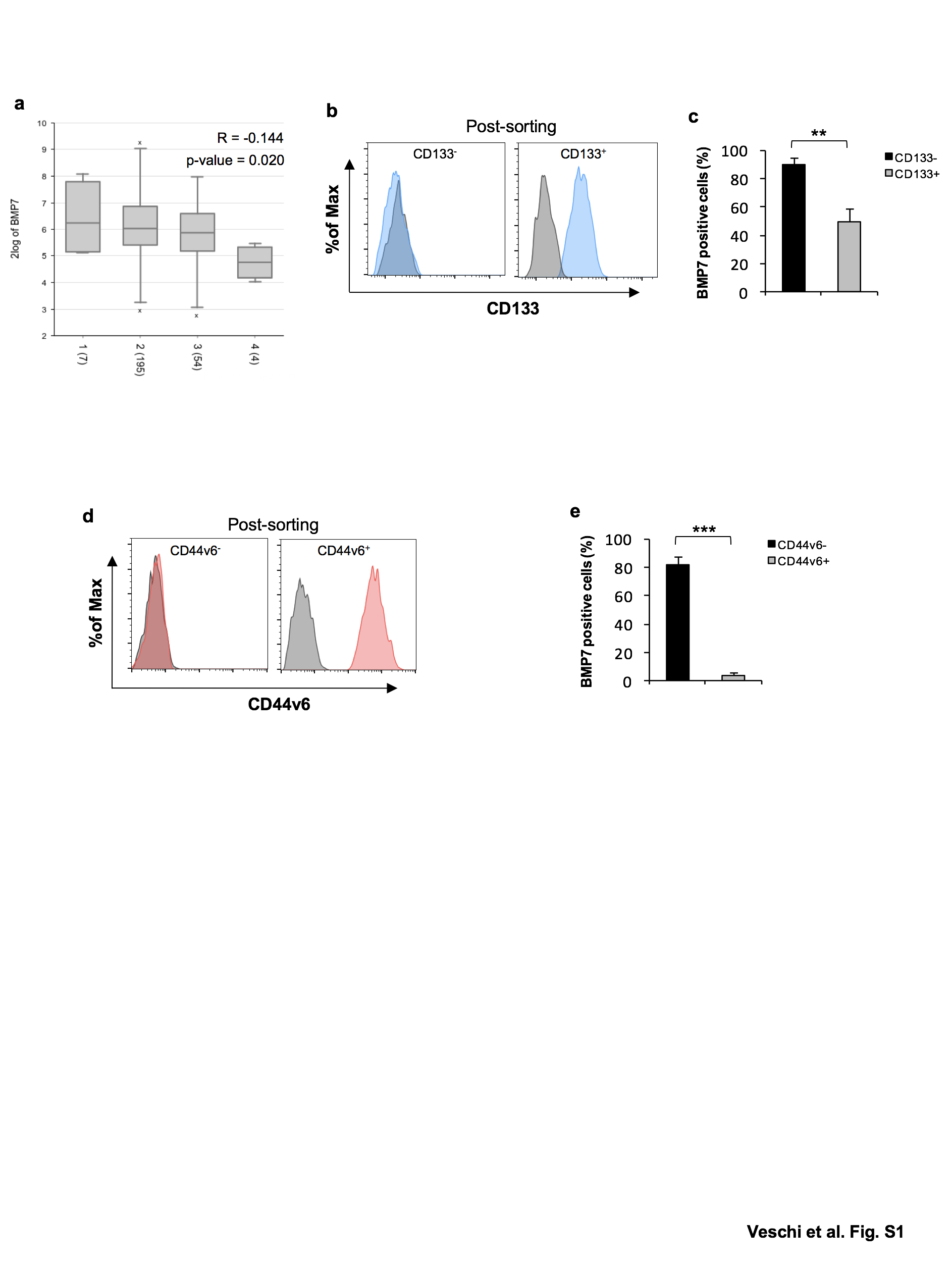

Supplement: Supplementary file 2 — Supplementary Figure 1 [file 41388_2019_1047_MOESM2_ESM.tif]

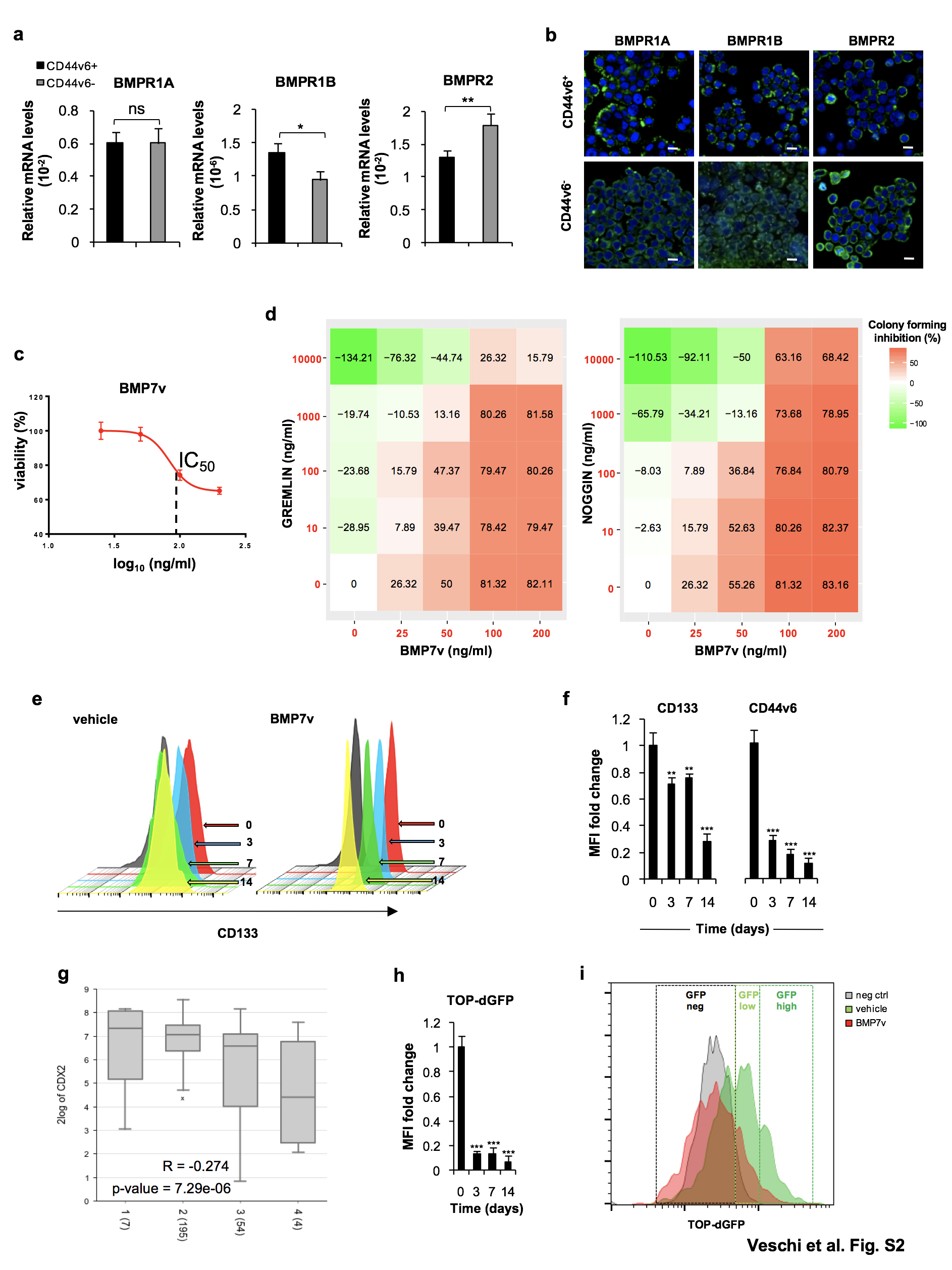

Supplement: Supplementary file 3 — Supplementary Figure 2 [file 41388_2019_1047_MOESM3_ESM.tif]

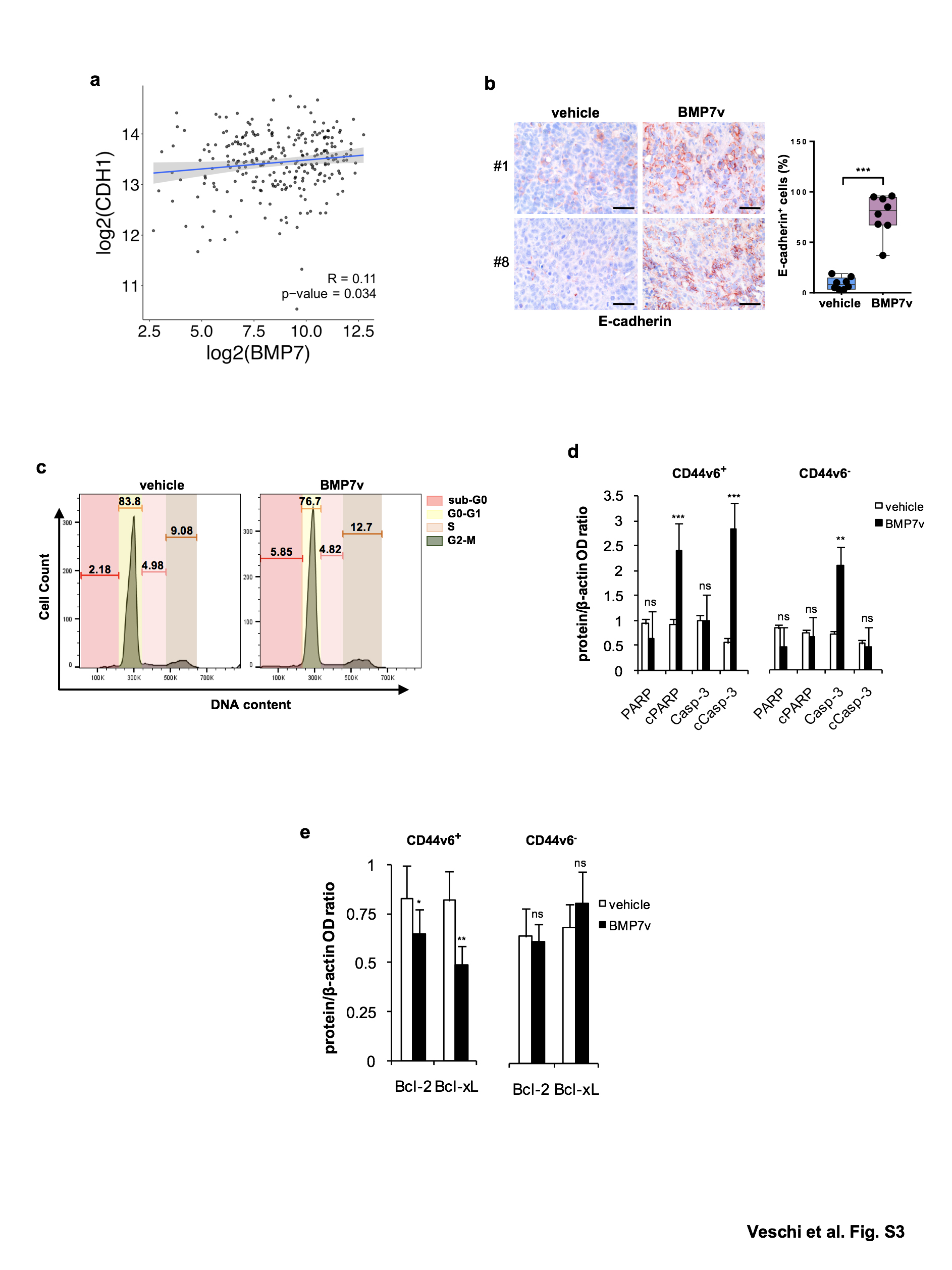

Supplement: Supplementary file 4 — Supplementary Figure 3 [file 41388_2019_1047_MOESM4_ESM.tif]

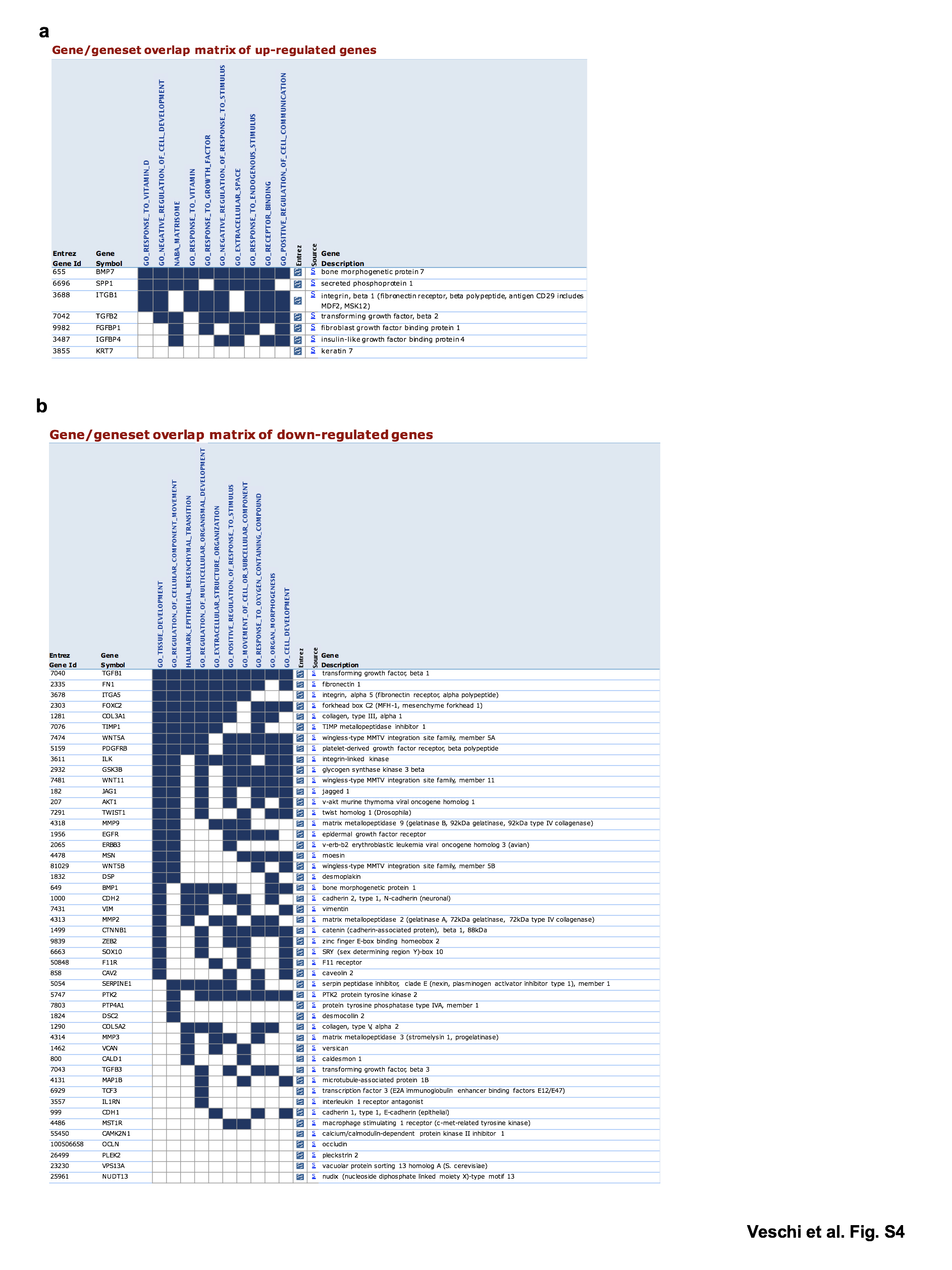

Supplement: Supplementary file 5 — Supplementary Figure 4 [file 41388_2019_1047_MOESM5_ESM.tif]

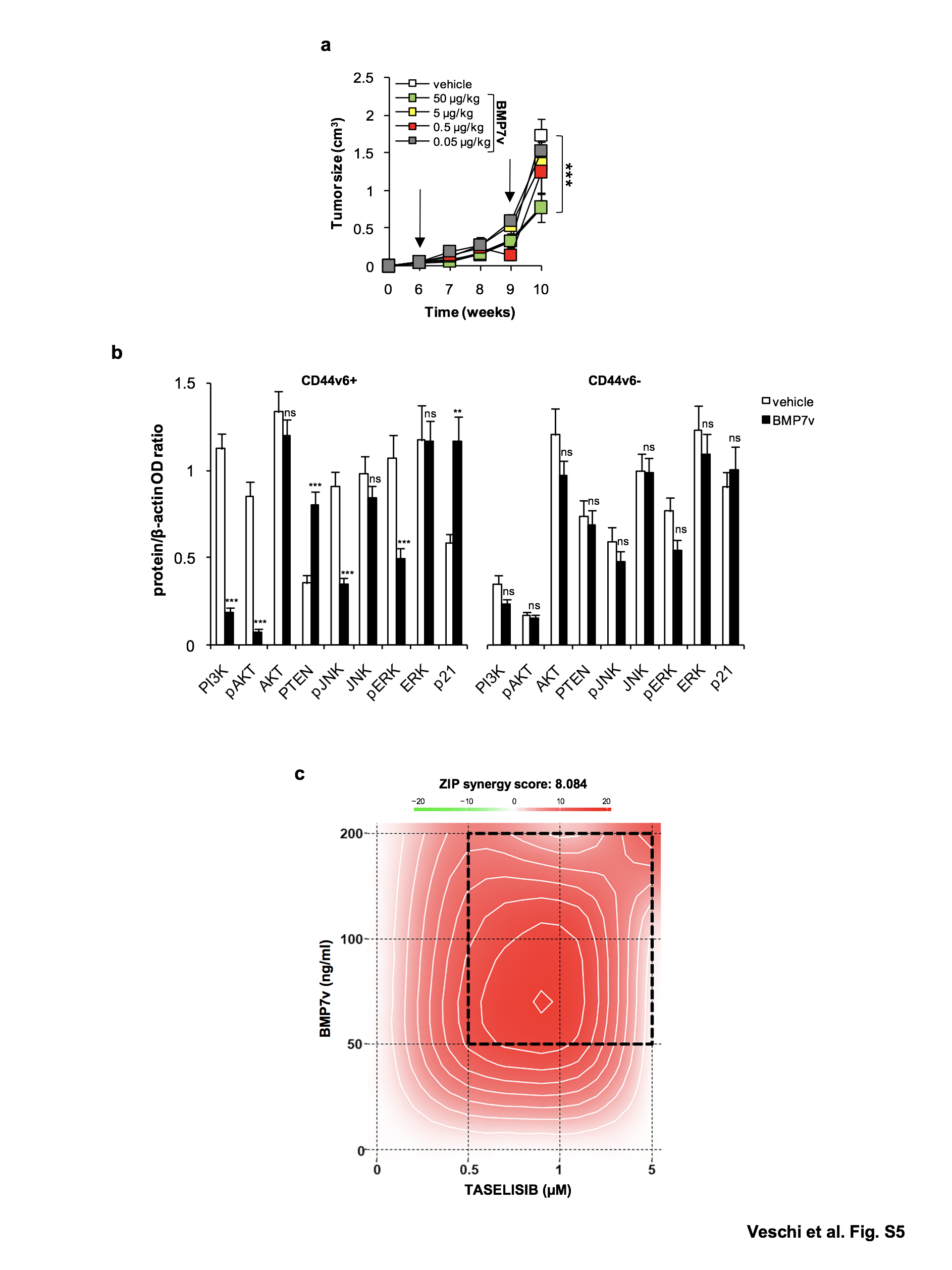

Supplement: Supplementary file 6 — Supplementary Figure 5 [file 41388_2019_1047_MOESM6_ESM.tif]
