# Supplementary material for: Targeting chemoresistant colorectal cancer via systemic administration of a BMP7 variant
Source: Oncogene. 2019 Oct 7;39(5):987–1003. doi: 10.1038/s41388-019-1047-4 (PMC6989400; doi:10.1038/s41388-019-1047-4)
Supplement: Supplementary file 3 [file 41388_2019_1047_MOESM7_ESM.docx]

Supplementary Table 1: Clinical data of CRC patients from which CRC sphere cell lines were derived.

CR-CSC#	Age/Gender	Site	Grade	TNM classification	Stage	Ethnicity
**1**	68/M	sigma	G3	T2N1M0	IIIA	Caucasian
**2**	41/M	left	G3	T3N0M0	IIA	Caucasian
**3**	85/F	right-ileum	G3	T3N2M0	IIIC	Caucasian
**4**	59/F	right	G2	T3N0M0	IIA	Caucasian
**5**	68/M	right	G3	T4N1M0	IIIB	Caucasian
**6**	76/M	right	G2	T2N1M0	IIIA	Caucasian
**7**	62/M	right	G2	T3N1M0	IIIB	Caucasian
**8**	57/F	caecum	G3	T3N2M1	IV	Caucasian
**9**	53/F	recto-sigmoid	G2	T3N2M1	IV	Caucasian
**10**	69/M	left	G3	T4N3M1	IV	Caucasian
**11**	77/F	right	G2	T3N1M0	IIIB	Caucasian
**12**	76/M	right	G2	T3N2M0	IIIC	Caucasian
**13**	63/M	right	G3	T4N2M0	IIIC	Caucasian
**14**	47/F	right	G2	T3N0M0	IIA	Caucasian
**15**	69/M	left	G2	T3N1M1	IV	Caucasian
**16**	79/F	right	G2	T3N0M0	IIA	Caucasian
**17**	75/M	left	G2	T2N0M0	I	Caucasian
**18**	57/F	left	G3	T3N2M1	IV	Caucasian
**19**	63/F	sigma	G2	T4N2M1	IV	Caucasian
**20**	68/M	right	G2	T3N1M0	IIIB	Caucasian
**21**	60/F	right	G2	T3N1MX	IIIB	Caucasian
**22**	71/F	transverse	G2	T3N0M0	IIA	Caucasian
**23**	78/M	transverse	G2	T3N0M0	IIA	Caucasian
**24**	51/F	right	G2	T3N0M0	IIA	Caucasian
**25**	60/M	left	G2	T3N2M0	IIIC	Caucasian
**26**	58/F	recto-sigmoid	G2	T2N0MX	I	Caucasian
**27**	60/M	left	G3	T3N2M0	IIIC	Caucasian
**31**	67/M	right	G2	T3N1M0	IIIB	Caucasian
**33**	58/M	right	G2	T2N0M0	I	Caucasian
**40**	49/M	recto-sigmoid	G2	T2N1M0	IIIA	Caucasian
**54**	66/F	recto-sigmoid	G2	T2N1M0	IIIA	Caucasian
**R1**	65/M	liver metastasis	n.d.	T3N0M0	IIA	Caucasian
**R2**	61/F	liver metastasis	n.d.	T3N1M1	IV	Caucasian
**R3**	62/F	liver metastasis	n.d.	T3N1M0	IIIB	Caucasian
**R4**	38/M	liver metastasis	n.d.	T4N1M1	IV	Caucasian
