# Supplementary material for: Targeting chemoresistant colorectal cancer via systemic administration of a BMP7 variant
Source: Oncogene. 2019 Oct 7;39(5):987–1003. doi: 10.1038/s41388-019-1047-4 (PMC6989400; doi:10.1038/s41388-019-1047-4)
Supplement: Supplementary file 4 [file 41388_2019_1047_MOESM8_ESM.docx]

**Supplementary Table 2**: COSMIC-reported mutations of *the indicated genes* and MSI profile, CD44v6 expression, CMS in CRC sphere cell lines.

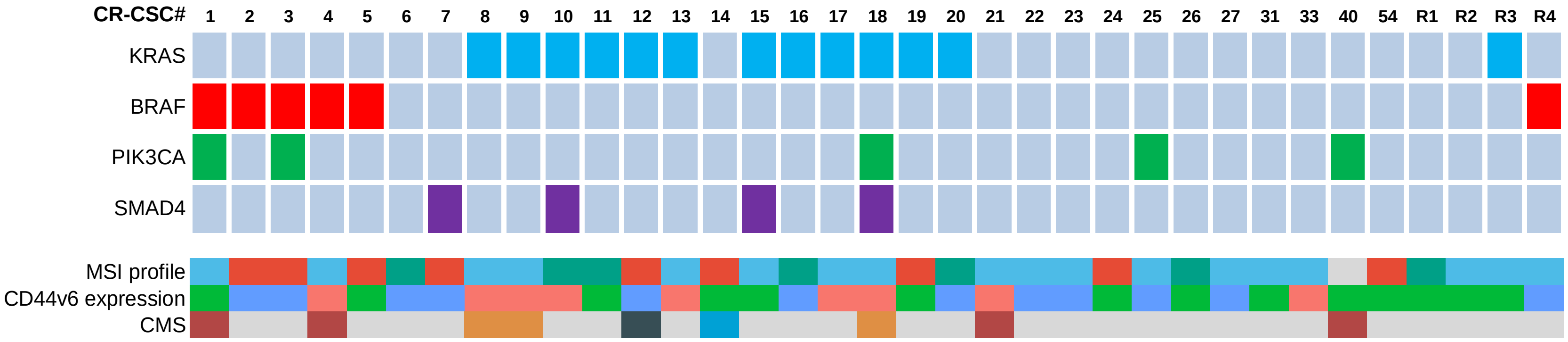


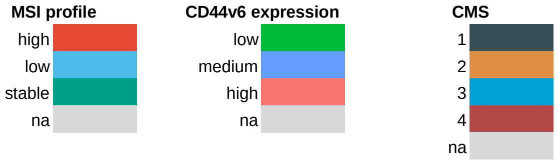


Bluish color indicates wild type genes in specific cell lines while blue, red, green and purple colors indicate KRAS, BRAF, PIK3CA and SMAD4 mutations, respectively. Percentage of mutation rate in CRC patients reported in COSMIC database (white bars) and in CRC sphere cell lines (black bars) is shown on the right.
